# *OsBADH1–OsBADH2* Double Mutants Increase 2-Acetyl-1-Pyrroline Accumulation and Alter GABA-Associated Abiotic Stress Responses in Rice

**DOI:** 10.3390/genes17050579

**Published:** 2026-05-18

**Authors:** Yu-Jin Jung, Jin-Young Kim, Kwon-Kyoo Kang

**Affiliations:** 1 Division of Horticultural Biotechnology, Hankyong National University, Anseong 17579, Republic of Korea; yuyu1216@hknu.ac.kr (Y.-J.J.); zino@hknu.ac.kr (J.-Y.K.); 2 Institute of Genetic Engineering, Hankyong National University, Anseong 17579, Republic of Korea

**Keywords:** rice, *OsBADH1*, *OsBADH2*, double mutant, 2-acetyl-1-pyrroline, GABA, abiotic stress

## Abstract

Background/Objectives: Rice fragrance is mainly determined by 2-acetyl-1-pyrroline (2-AP), which is negatively regulated by *OsBADH2*. However, the contribution of its paralog *OsBADH1* to aroma-associated metabolism and GABA-linked abiotic stress responses remains unclear. This study investigated whether simultaneous disruption of *OsBADH1* and *OsBADH2* further enhances 2-AP accumulation while affecting stress tolerance in rice. Methods: Independent *osbadh1* and *osbadh2* knockout lines were generated using CRISPR/Cas9 and crossed to obtain homozygous *osbadh1 osbadh2* double mutants. Wild type, single mutants, and double mutants were compared for 2-AP accumulation, GABA content, agronomic traits, abiotic stress responses, and expression of genes associated with GABA metabolism and stress responses. Results: The *osbadh2* mutant showed a marked increase in 2-AP, and the *osbadh1 osbadh2* double mutant exhibited the highest level, corresponding to a 7.1-fold increase over the wild type. In contrast, the GABA content progressively decreased and reached 0.46-fold of the wild-type level in the double mutant. Under normal growth conditions, the double mutant showed no major agronomic defects. However, under salinity and drought stress, its survival declined to 0.41-fold and 0.40-fold of the wild-type levels, respectively. KEGG and expression analyses further indicated coordinated disruption of GABA-associated metabolic and stress-responsive pathways in the double mutant. Conclusions: Combined disruption of *OsBADH1* and *OsBADH2* enhanced aroma-associated metabolism but weakened GABA-linked abiotic stress tolerance, revealing a trade-off between increased fragrance and reduced stress resilience in rice.

## 1. Introduction

Rice (*Oryza sativa* L.) is one of the world’s most important staple crops, and grain quality remains a major target in rice improvement programs. Among quality traits, fragrance is particularly valuable because it strongly influences consumer preference and market price. The characteristic aroma of fragrant rice is primarily attributed to 2-acetyl-1-pyrroline (2-AP), and loss of function of *OsBADH2* has been established as the major genetic basis of this trait [1,2,3,4]. Previous genetic and molecular studies identified *BADH2* as the key gene underlying rice fragrance, while allelic and breeding analyses demonstrated that nonfunctional *badh2* alleles are broadly associated with aromatic germplasm across diverse rice populations [1,2,3,4]. More recently, CRISPR/Cas9-based studies confirmed that targeted disruption of *OsBADH2* is an efficient strategy for generating fragrant rice lines, further highlighting its value in aroma-oriented rice improvement [5,6]. However, focusing on *OsBADH2* alone is unlikely to fully explain the biological consequences of fragrance-associated BADH disruption in rice. Rice contains a closely related paralog, *OsBADH1*, and available biochemical evidence indicates that the two BADH proteins are functionally distinct rather than redundant. OsBADH1 shows efficient acetaldehyde oxidation activity, whereas OsBADH2 exhibits extremely low activity toward the same substrate [7]. This distinction is biologically important because aldehyde dehydrogenases contribute to reactive aldehyde detoxification, metabolic homeostasis, and cellular protection under stress conditions. Thus, disruption of *OsBADH1* in an *osbadh2* background is not merely an additional mutation in a homologous gene; rather, it provides a genetic framework to test whether loss of an aldehyde-buffering pathway exacerbates the metabolic and physiological consequences associated with fragrance-related BADH perturbation [7]. This question is particularly relevant in the context of 2-AP biosynthesis.

Current models indicate that functional BADH2 converts γ-aminobutyraldehyde (GABald) into γ-aminobutyric acid (GABA), thereby limiting the formation of Δ^1^-pyrroline and downstream 2-AP accumulation [2]. When BADH2 is disrupted, this conversion is reduced, metabolic flux is redirected toward 2-AP-related intermediates, and aroma formation is enhanced [2,8,9]. Importantly, recent studies indicate that 2-AP biosynthesis is embedded within a broader metabolic network involving proline, ornithine, glutamate, GABA, and environmentally responsive metabolic regulation [8,9,10]. These findings suggest that fragrance-associated BADH disruption affects not only volatile formation but also broader metabolic processes linked to aldehyde turnover, nitrogen balance, and stress acclimation [8,9,10]. GABA provides a key link between aroma-associated metabolism and stress physiology. In plants, GABA is widely implicated in responses to salinity, drought, heat, and oxidative stress, with functions in redox homeostasis, carbon–nitrogen coordination, osmotic adjustment, and stress-related signaling [11,12,13]. In fragrant rice, the relationship among BADH2, GABA-associated metabolism, and 2-AP accumulation has been discussed extensively, but most evidence remains centered on *OsBADH2* alone or on comparisons between aromatic and non-aromatic backgrounds [2,8,9,10]. Whether simultaneous disruption of *OsBADH1* and *OsBADH2* further redirects metabolic flux toward aroma while weakening GABA-associated buffering capacity has not been directly tested. This unresolved question is important because it addresses whether fragrance-oriented gene editing may confer not only quality benefits but also a physiological cost under adverse environmental conditions.

In this study, we generated independent *osbadh1* and *osbadh2* knockout lines using CRISPR/Cas9 and crossed the single mutants to produce homozygous *osbadh1 osbadh2* double mutants. By comparing wild type, single mutants, and double mutants, we examined the effects of combined *OsBADH1* and *OsBADH2* disruption on 2-AP accumulation, GABA content, agronomic traits, and responses to salinity and drought stress. We hypothesized that *OsBADH1* and *OsBADH2* make genetically distinct but interacting contributions to BADH-associated metabolism and that simultaneous loss of both genes would enhance aroma-associated metabolic reprogramming beyond the effect of *OsBADH2* alone while increasing abiotic stress sensitivity. By linking fragrance-related metabolism with stress physiology, this study provides a genetic and physiological framework for evaluating the trade-off between aroma improvement and stress resilience in rice breeding.

## 2. Materials and Methods

### 2.1. Plant Materials and Growth Conditions

Rice (*O. sativa* L. cv. Dongjin) was used in this study. Wild-type and mutant plants were grown in a controlled greenhouse under a 12 h light/12 h dark photoperiod at 28 °C during the day and 25 °C at night. For routine growth, plants were cultivated in soil until maturity for phenotypic and agronomic evaluation. For abiotic stress assays, seedlings were germinated and transferred to hydroponic culture, and 2-week-old seedlings were used for stress treatments. Hydroponic cultivation was performed according to the standard rice culture solution procedure described by Yoshida [14].

### 2.2. CRISPR/Cas9 Vector Construction and Plant Transformation

RISPR/Cas9 target sites for *OsBADH1* and *OsBADH2* were selected within coding regions using Cas-Designer [15], which is available through the CRISPR RGEN Tools platform (http://www.rgenome.net/cas-designer/; accessed on 21 April 2024) (Supplementary Table S1). Candidate sgRNAs were further evaluated for potential off-target sites using Cas-OFFinder [16] in the same platform (http://www.rgenome.net/cas-offinder/; accessed on 21 April 2024). For each sgRNA, potential off-target sites in the rice genome were searched by allowing one, two, or three mismatches relative to the target sequence. Under these search conditions, only the intended target site was detected for each selected sgRNA, and no additional genomic sites with one to three mismatches were identified. The sgRNA design information and mismatch-based off-target prediction results are summarized in Supplementary Table S1. Complementary oligonucleotides corresponding to each sgRNA were annealed and cloned into the pBOsC CRISPR/Cas9 binary vector under the control of the rice U3 promoter (Supplementary Figure S1A). The inserted sgRNA regions were confirmed by Sanger sequencing prior to plant transformation. The confirmed constructs were introduced into *Agrobacterium tumefaciens* strain EHA105. Mature seeds of Dongjin were used to induce embryogenic calli (Supplementary Figure S1B). Actively growing calli were infected with *Agrobacterium* harboring each construct and co-cultivated according to the *Agrobacterium*-mediated rice transformation protocol described by Nishimura et al. [17]. After co-cultivation, infected calli were transferred to selection medium, and resistant calli were regenerated into shoots and rooted to obtain independent T0 plants. Regenerated plants were screened for targeted mutations according to previously reported rice CRISPR/Cas9 procedures [18,19].

### 2.3. Genotyping and Mutation Analysis

Genomic DNA was extracted from young leaves using a CTAB-based method [20]. Target regions flanking each sgRNA site were amplified by PCR using gene-specific primers (Supplementary Table S2). PCR products were purified and subjected to Sanger sequencing to identify insertion/deletion mutations. Mutation types were determined by comparison with the wild-type genomic sequence, and homozygous knockout lines were selected for further analysis [18,19]. Sequence chromatograms obtained by Sanger sequencing were analyzed to determine the type and size of edited alleles, and mutation patterns were interpreted according to the general analytical framework used for CRISPR/Cas9-induced indel analysis, including chromatogram-based sequence assessment, as described in Cas-Analyzer (http://www.rgenome.net/cas-analyzer/; accessed on 17 September 2024) [21]. To confirm segregation of the CRISPR/Cas9 T-DNA, later-generation plants carrying homozygous edited alleles were screened by PCR using T-DNA-specific primers targeting the vector backbone/selectable marker region. Plants showing the expected edited alleles at the *OsBADH1* and/or *OsBADH2* loci but lacking amplification of the T-DNA-specific fragment were selected as transgene-free mutant lines and used for subsequent biochemical, physiological, and gene expression analyses. The primers used for T-DNA confirmation are listed in Supplementary Table S2.

### 2.4. Generation of Double Mutants

Homozygous *osbadh1* and *osbadh2* single-mutant lines carrying frameshift-inducing deletions predicted to generate premature stop codons were selected as parental lines for crossing. The selected *osbadh1* and *osbadh2* lines were crossed to generate F_1_ plants. F_1_ individuals were self-pollinated, and F_2_ populations were screened by PCR-based genotyping and sequencing to identify homozygous *osbadh1 osbadh2* double mutants. For each selected F_2_ line, the edited status of both loci was confirmed by sequence analysis. Segregation of the edited alleles in the F_2_ generation was evaluated according to the expected Mendelian ratio.

### 2.5. 2-Acetyl-1-Pyrroline and GABA Analysis

For 2-acetyl-1-pyrroline (2-AP) analysis, mature rice grains were harvested, dehulled, immediately frozen in liquid nitrogen, and ground into a fine powder. Volatile compounds were collected from the powdered grain matrix using headspace solid-phase microextraction (HS-SPME), following previously reported approaches for volatile profiling and aroma compound analysis in fragrant and non-fragrant rice [22,23]. Briefly, 4.0 g of powdered grain sample was placed in a 20 mL sealed headspace vial, equilibrated at 80 °C for 30 min, and extracted using a 75 μm CAR/PDMS SPME fiber for 60 min. GC–MS analysis was performed using an Agilent 7890A gas chromatograph coupled to an Agilent 5975C inert XL mass-selective detector (Agilent Technologies, Santa Clara, CA, USA). Volatiles were separated on a DB-5 capillary column (30 m × 0.25 mm × 0.25 μm) using helium as the carrier gas at 1.0 mL min^−1^. The GC oven temperature was programmed as follows: 40 °C for 3 min, increased to 100 °C at 5 °C min^−1^ and held for 3 min, then increased to 250 °C at 10 °C min^−1^ and held for 4 min. 2-AP was identified by comparison of retention time and mass spectrum with an authentic standard and quantified using standard calibration curves. Raw data for 2-AP analysis are provided in Supplementary Table S3. For GABA analysis, mature rice grain samples were homogenized and extracted, and the extracts were subjected to derivatization prior to chromatographic separation. GABA content was determined by high-performance liquid chromatography (HPLC) after derivatization, following previously described procedures for GABA quantification in rice samples [24,25]. Quantification was based on calibration curves generated with authentic GABA standards. Raw data for GABA analysis are provided in Supplementary Table S4.

### 2.6. Phenotypic and Agronomic Analysis

Phenotypic and agronomic traits were evaluated at the mature stage according to the Standard Evaluation System for Rice of the International Rice Research Institute [26]. Plant height was measured from the soil surface to the tip of the tallest panicle, excluding awns. Tiller number was recorded as the total number of tillers per plant. Panicle-related traits were evaluated using representative main panicles harvested at maturity and included panicle length and grain set. Grain-related traits were assessed after harvest using fully matured seeds. Where applicable, seed morphology was examined using representative grains from each genotype. Quantitative measurements were collected from independent biological replicates for each genotype and used for genotype-based comparisons.

### 2.7. Abiotic Stress Treatment and Physiological Analysis

For salt stress treatment, 2-week-old hydroponically grown seedlings were transferred to a nutrient solution containing 150 mM NaCl and maintained for 7 days [27]. For drought stress treatment, soil-grown plants were subjected to water withdrawal until visible wilting occurred, followed by re-watering to assess recovery [28]. Stress symptoms were photographed, and survival rates were recorded after treatment. Relative water content was determined according to the method of Barrs and Weatherley [29]. Briefly, fresh weight was measured immediately after sampling, leaves were rehydrated to obtain turgid weight, and samples were then dried to determine dry weight. Chlorophyll content was measured spectrophotometrically according to Arnon [30] using a UV-1800 spectrophotometer (Shimadzu, Kyoto, Japan). Additional physiological parameters associated with stress responses were evaluated as indicated in Supplementary Table S5.

### 2.8. Gene Expression and Statistical Analysis

Total RNA was extracted from plant tissues using TRIzol reagent (Invitrogen, Carlsbad, CA, USA) according to the manufacturer’s instructions. Reverse transcription was performed using the SuPrimeScript RT premix kit (Genet Bio, Daejeon, Republic of Korea). Quantitative real-time PCR (qRT-PCR) was carried out using gene-specific primers, and relative expression levels were calculated using the 2^−ΔΔCt^ method [31]. *OsActin* was used as the internal reference gene. Primer information is provided in Supplementary Table S2. KEGG pathway-based analysis was performed using the targeted stress-associated gene expression dataset and metabolite profiling dataset generated in this study. This analysis was used to interpret pathway-level changes associated with GABA metabolism, amino acid metabolism, aldehyde-related metabolism, and stress responses.

### 2.9. Statistical Analysis

All experiments were conducted with at least three biological replicates. Data are presented as mean ± standard deviation (SD). Statistical significance was determined by one-way analysis of variance (ANOVA), followed by Tukey’s multiple comparison test. Differences were considered significant at *p* < 0.05.

## 3. Results

### 3.1. Generation of osbadh1, osbadh2, and osbadh1 osbadh2 Double Mutants

**Figure 1:**
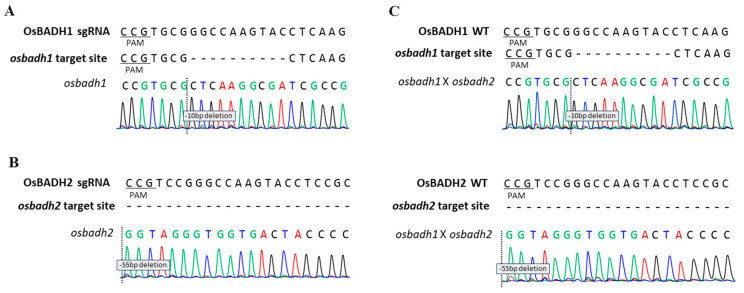
Sanger sequencing-based confirmation of homozygous *osbadh1*, *osbadh2*, and *osbadh1 osbadh2* double mutants. (**A**) Wild-type and homozygous edited alleles of *OsBADH1*. (**B**) Wild-type and homozygous edited alleles of *OsBADH2*. (**C**) Confirmation of the homozygous *osbadh1 osbadh2* double mutant obtained by crossing the homozygous *osbadh1* and *osbadh2* single-mutant lines. The double mutant carried edited alleles at both *OsBADH1* and *OsBADH2* loci.

To investigate the genetic relationship between *OsBADH1* and *OsBADH2*, independent knockout lines for each gene were generated using the CRISPR/Cas9 system. Target sites were designed within the coding regions of *OsBADH1* and *OsBADH2*, and regenerated plants were screened by PCR and sequence analysis (Supplementary Table S6 and Figure S1C). Multiple edited alleles with distinct mutation patterns were identified, and homozygous mutant lines were obtained through self-pollination of the edited plants. Among these, representative alleles carrying a 10 bp deletion in *OsBADH1* and a 55 bp deletion in *OsBADH2* were selected for further analysis (Figure 1A,B; Supplementary Figure S1). Hereafter, the line carrying the 10 bp deletion in *OsBADH1* is referred to as *osbadh1*, and the line carrying the 55 bp deletion in *OsBADH2* is referred to as *osbadh2*. Both mutations were predicted to cause frameshifts leading to premature termination codons, indicating loss of normal *OsBADH1* or *OsBADH2* gene function (Supplementary Figure S2). To generate the double mutant, the selected homozygous *osbadh1* and *osbadh2* single-mutant lines were crossed, and the resulting F1 plants were self-pollinated. F2 progeny were subsequently screened by PCR-based genotyping and sequence analysis, which identified homozygous *osbadh1 osbadh2* double-mutant individuals carrying edited alleles at both loci (Figure 1C). Segregation of the edited alleles in the F2 population generally followed the expected Mendelian pattern (Supplementary Table S7). In later generations, the selected homozygous *osbadh1*, *osbadh2*, and *osbadh1 osbadh2* mutant lines were further screened by PCR using T-DNA-specific primers, and only lines lacking CRISPR/Cas9 T-DNA-specific amplification were used for subsequent analyses (Supplementary Figure S3B). These results confirmed the successful establishment of transgene-free *osbadh1*, *osbadh2*, and *osbadh1 osbadh2* mutant lines for subsequent analyses of 2-AP accumulation, GABA content, and abiotic stress responses.

### 3.2. Enhanced 2-AP Accumulation in Double Mutants

**Figure 2:**
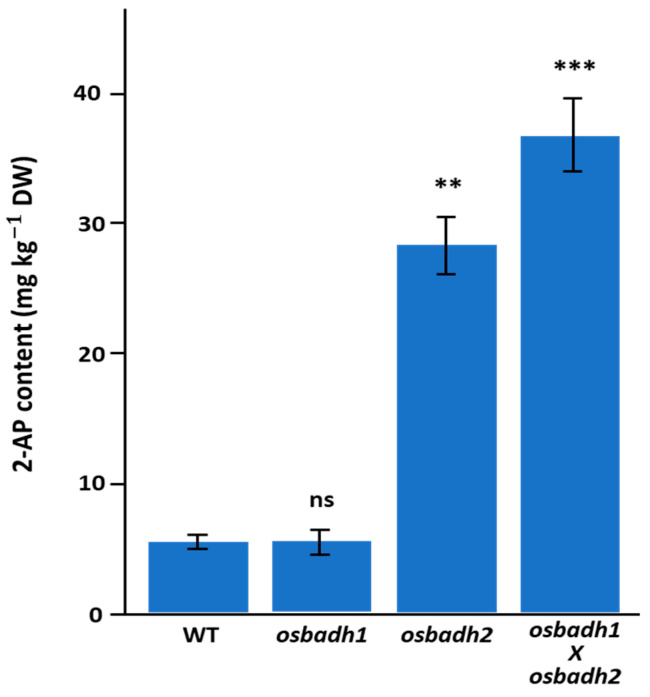
2-AP content in the wild type, *osbadh1*, *osbadh2*, and *osbadh1 osbadh2* double mutant. Data are presented as mean ± SD. Statistical significance was evaluated relative to the wild type; ns, not significant; ** *p* < 0.01; *** *p* < 0.001.

To assess whether combined disruption of *OsBADH1* and *OsBADH2* further enhances aroma-related metabolite accumulation, 2-acetyl-1-pyrroline (2-AP) content was measured in the wild type, *osbadh1*, *osbadh2*, and the *osbadh1 osbadh2* double mutant. The *osbadh1* mutant showed a 2-AP level of 5.5 ± 0.9 mg kg^−1^ DW, which was comparable to that of the wild type (5.2 ± 0.8 mg kg^−1^ DW) and was not significantly different. In contrast, the *osbadh2* mutant exhibited a marked increase in 2-AP accumulation, reaching 28.4 ± 2.1 mg kg^−1^ DW, corresponding to a 5.5-fold increase relative to the wild type (*p* < 0.01). Notably, the *osbadh1 osbadh2* double mutant showed the highest 2-AP content (36.7 ± 2.8 mg kg^−1^ DW), representing a 7.1-fold increase compared with the wild type (*p* < 0.001) (Figure 2). Consistent with these results, the relative 2-AP levels were 1.0 in the wild type, 1.1 in *osbadh1*, 5.5 in *osbadh2*, and 7.1 in the double mutant. Together, these findings indicate that *OsBADH2* is the principal determinant of elevated 2-AP accumulation, while additional disruption of *OsBADH1* further enhances 2-AP accumulation in the *osbadh2* background.

### 3.3. Reduction of GABA and Related Metabolites in Double Mutants

**Figure 3:**
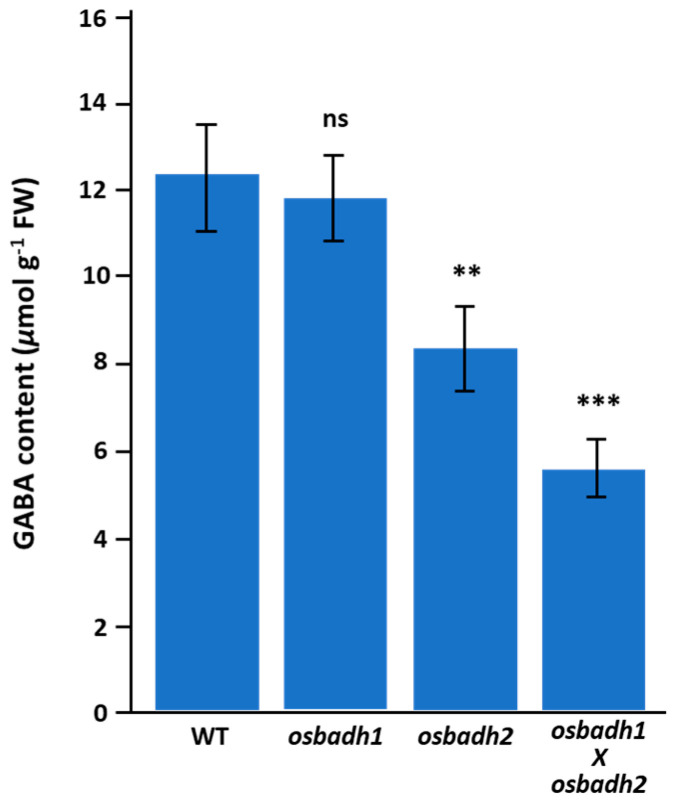
GABA content in the wild type, *osbadh1*, *osbadh2*, and *osbadh1 osbadh2* double mutant. Data are presented as mean ± SD. Statistical significance was evaluated relative to the wild type; ns, not significant; ** *p* < 0.01; *** *p* < 0.001.

To examine the effect of *OsBADH1* and *OsBADH2* disruption on GABA-associated metabolism, γ-aminobutyric acid (GABA) content was quantified in the wild type, *osbadh1*, *osbadh2*, and the *osbadh1 osbadh2* double mutant. The *osbadh1* mutant showed a GABA content of 11.8 ± 1.0 μmol g^−1^ FW, which was comparable to that of the wild type (12.3 ± 1.2 μmol g^−1^ FW) and was not significantly different. In contrast, the *osbadh2* mutant exhibited a significant reduction in GABA content to 8.4 ± 0.9 μmol g^−1^ FW, corresponding to 0.68-fold of the wild type (*p* < 0.01). Notably, the *osbadh1 osbadh2* double mutant showed the lowest GABA level (5.6 ± 0.7 μmol g^−1^ FW), representing 0.46-fold of the wild type (*p* < 0.001) (Figure 3; Supplementary Table S4). Consistent with these results, the relative GABA levels were 1.0 in the wild type, 0.96 in *osbadh1*, 0.68 in *osbadh2*, and 0.46 in the double mutant. Metabolite profiling further showed that GABA- and 2-AP-related metabolites were altered in a genotype-dependent manner (Supplementary Table S4). Glutamate, proline, and γ-aminobutyraldehyde were reduced in the *osbadh2* mutant and showed further decreases in the *osbadh1 osbadh2* double mutant. In contrast, 2-AP accumulation was markedly increased in the *osbadh2* mutant and reached the highest level in the double mutant. Together, these findings indicate that disruption of *OsBADH2* reduces GABA-associated metabolite accumulation, while additional disruption of *OsBADH1* further shifts the metabolic balance toward enhanced 2-AP accumulation in the *osbadh2* background.

### 3.4. Normal Growth Under Non-Stress Conditions

To determine whether disruption of *OsBADH1* and *OsBADH2* affects plant growth and agronomic performance under normal conditions, the wild type, *osbadh1*, *osbadh2*, and the *osbadh1 osbadh2* double mutant were compared for major vegetative and yield-related traits. Plant height was 102.5 ± 4.3 cm in the wild type, 101.8 ± 5.0 cm in *osbadh1*, 100.6 ± 4.7 cm in *osbadh2*, and 99.8 ± 5.2 cm in the double mutant. Likewise, tiller number per plant was 12.1 ± 1.2, 11.8 ± 1.4, 11.5 ± 1.3, and 11.3 ± 1.5, respectively. Panicle length also showed only minor variation among genotypes, with values of 23.4 ± 1.1 cm in the wild type, 23.1 ± 1.3 cm in *osbadh1*, 22.9 ± 1.2 cm in *osbadh2*, and 22.7 ± 1.4 cm in the double mutant. Grain-related traits were similarly comparable among the genotypes. Grain number per panicle was 128.6 ± 8.7 in the wild type, 125.9 ± 7.9 in *osbadh1*, 122.3 ± 9.1 in *osbadh2*, and 120.7 ± 8.5 in the double mutant, whereas 1,000-grain weight was 24.8 ± 0.9 g, 24.5 ± 1.0 g, 24.2 ± 0.8 g, and 24.0 ± 0.9 g, respectively. None of these differences were statistically significant (Table 1 and Supplementary Figure S3). These results indicate that single or combined disruption of *OsBADH1* and *OsBADH2* does not cause marked defects in plant growth or agronomic performance under non-stress conditions.

### 3.5. Reduced Abiotic Stress Tolerance in Double Mutants

**Figure 4:**
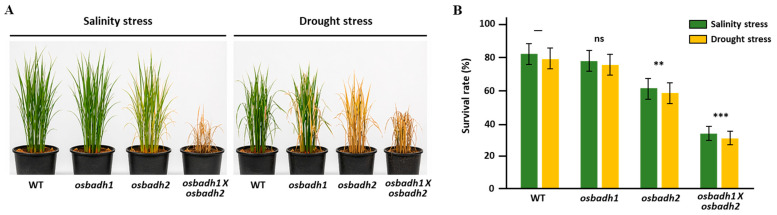
Abiotic stress responses of the wild type, *osbadh1*, *osbadh2*, and osbadh1 osbadh2 double mutant. (**A**) Representative seedling phenotypes under salinity and drought stress conditions. (**B**) Relative survival rates after stress treatments. Data are presented as mean ± SD from three independent biological replicates. Statistical significance was evaluated relative to the wild type; ns, not significant; ** *p* < 0.01; *** *p* < 0.001.

Given that the *osbadh1 osbadh2* double mutant exhibited enhanced 2-AP accumulation together with reduced GABA content under normal conditions, we next examined whether these metabolic differences were associated with altered abiotic stress responses. The wild type, *osbadh1*, *osbadh2*, and the *osbadh1 osbadh2* double mutant were subjected to salinity and drought treatments. Representative phenotypes showed that *osbadh2* and, more prominently, the *osbadh1 osbadh2* double mutant exhibited more severe stress symptoms than the wild type and *osbadh1* under both conditions, including enhanced chlorosis, wilting, and growth inhibition (Figure 4A). Consistent with these observations, survival assays revealed a marked reduction in stress tolerance in the mutants carrying the *osbadh2* allele. Under salinity stress, survival rates were 82.3 ± 4.8% in the wild type, 78.5 ± 5.1% in *osbadh1*, 61.4 ± 4.3% in *osbadh2*, and 34.2 ± 3.6% in the *osbadh1 osbadh2* double mutant. A similar pattern was observed under drought stress, with survival rates of 79.6 ± 5.2%, 75.2 ± 4.9%, 58.7 ± 4.6%, and 31.8 ± 3.9%, respectively. Relative survival was 1.0 in the wild type, 0.95 in *osbadh1*, 0.74 in *osbadh2*, and 0.41 in the double mutant. Statistical analysis showed no significant difference in *osbadh1*, whereas survival was significantly reduced in *osbadh2* (*p* < 0.01) and further reduced in the *osbadh1 osbadh2* double mutant (*p* < 0.001) compared with the wild type (Figure 4B). Physiological measurements further supported the increased stress sensitivity of the double mutant. Relative water content decreased from 82.6 ± 3.5% in the wild type to 68.7 ± 3.1% in *osbadh2* and 55.3 ± 2.8% in the double mutant, while chlorophyll content declined from 38.4 ± 2.1 SPAD in the wild type to 32.5 ± 2.0 and 27.8 ± 1.9 SPAD, respectively. In contrast, stress-associated damage indicators increased progressively in the mutants, with electrolyte leakage rising from 18.2 ± 2.3% in the wild type to 28.6 ± 3.0% in *osbadh2* and 41.2 ± 3.4% in the double mutant. Likewise, MDA content increased from 2.8 ± 0.3 to 4.5 ± 0.5 and 6.8 ± 0.6 nmol g^−1^ FW, and ROS levels increased from 1.0 to 1.6 and 2.3 relative units, respectively (Supplementary Table S5). Together, these results indicate that mutants carrying the *osbadh2* allele, particularly the *osbadh1 osbadh2* double mutant, are more susceptible to salinity and drought stress than the wild type and *osbadh1*.

### 3.6. Altered GABA-Associated Stress Pathways in Double Mutants

**Figure 5:**
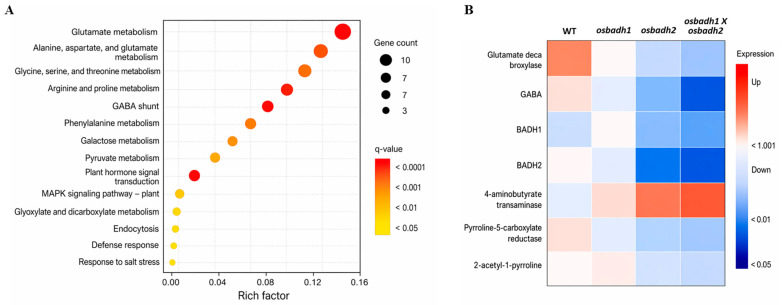
KEGG pathway-based analysis and heat map of GABA-associated stress pathways in rice BADH mutants. (**A**) KEGG pathway-based analysis using targeted stress-associated gene expression and metabolite profiling datasets generated in this study. (**B**) Heat map showing expression patterns of genes associated with GABA metabolism, stress responses, and aldehyde detoxification in WT and mutant lines.

**Figure 6:**
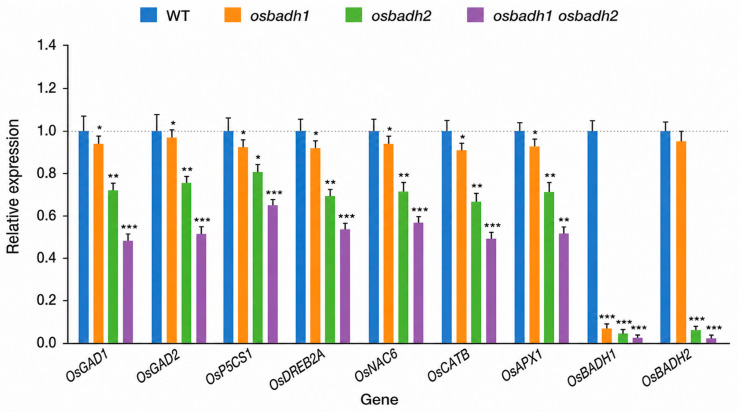
Relative expression levels of selected GABA metabolism-, stress response-, and aldehyde detoxification-related genes in rice *BADH* mutants. Expression levels of *OsGAD1*, *OsGAD2*, *OsP5CS1*, *OsDREB2A*, *OsNAC6*, *OsCATB*, *OsAPX1*, *OsBADH1*, and *OsBADH2* in WT, *osbadh1*, *osbadh2*, and *osbadh1 osbadh2* mutants by qRT-PCR. Data are presented as mean ± SD from three biological replicates. Asterisks indicate significant differences compared with WT (* *p* < 0.05, ** *p* < 0.01, *** *p* < 0.001).

To investigate the molecular basis underlying the increased abiotic stress sensitivity of the mutants, we performed KEGG pathway-based analysis using the targeted stress-associated gene expression dataset and metabolite profiling dataset generated in this study. The affected pathways were mainly associated with GABA-related amino acid metabolism and stress responses, including glutamate metabolism, alanine, aspartate and glutamate metabolism, arginine and proline metabolism, the GABA shunt, plant hormone signal transduction, and MAPK signaling (Figure 5A). To further examine these pathway-level changes, we analyzed the expression patterns of genes associated with GABA metabolism, stress responses, and aldehyde detoxification. The heatmap showed a clear overall shift in expression profiles across the mutants, with the most pronounced changes observed in the *osbadh1 osbadh2* double mutant (Figure 5B). Consistent with this pattern, targeted expression analysis showed that *OsGAD1* and *OsGAD2* were reduced to 0.48 ± 0.05-fold and 0.51 ± 0.06-fold of wild-type levels, respectively, in the double mutant (Figure 6). Likewise, *OsP5CS1*, *OsDREB2A*, and *OsNAC6* were downregulated to 0.65 ± 0.05-fold, 0.54 ± 0.05-fold, and 0.57 ± 0.05-fold, respectively, while *OsCATB* and *OsAPX1* decreased to 0.49 ± 0.05-fold and 0.52 ± 0.04-fold, respectively. As expected, *OsBADH1* transcript levels were markedly reduced in the *osbadh1* single mutant and in the *osbadh1 osbadh2* double mutant, whereas *OsBADH2* transcript levels were markedly reduced in the *osbadh2* single mutant and in the double mutant. In the double mutant, *OsBADH1* and *OsBADH2* transcript levels decreased to 0.04 ± 0.01-fold and 0.02 ± 0.01-fold of wild-type levels, respectively. Together, these results indicate that simultaneous disruption of *OsBADH1* and *OsBADH2* disturbs GABA-associated metabolic homeostasis and weakens stress-responsive pathways, which is consistent with the reduced GABA accumulation and enhanced abiotic stress sensitivity observed in the double mutant.

## 4. Discussion

Rice fragrance has classically been interpreted through the BADH2–2-AP axis, but current evidence indicates that aroma formation is embedded in a broader metabolic framework involving aldehyde turnover, amino acid metabolism, GABA homeostasis, and abiotic stress adaptation [32,33,34,35,36]. Within this framework, the present study examined whether simultaneous disruption of *OsBADH1* and *OsBADH2* would intensify aroma-associated metabolic reprogramming beyond the effect of *OsBADH2* alone and whether such reprogramming would impose a physiological cost. Our results support this interpretation: the *osbadh1 osbadh2* double mutant showed the highest 2-AP accumulation (Figure 2), the lowest GABA content (Figure 3; Supplementary Table S4), and the strongest sensitivity to salinity and drought stress (Figure 4; Supplementary Table S5). The stronger aroma phenotype of the double mutant indicates that the metabolic consequences of BADH disruption cannot be explained solely by *OsBADH2* disruption. Previous genetic, biochemical, and physiological studies established that loss of BADH2 activity promotes fragrance by favoring 2-AP formation, and several reports further showed that fragrance-associated metabolism is closely linked to precursor availability, aldehyde handling, and stress-responsive metabolic regulation [32,33,37,38,39,40]. In this context, the further increase in 2-AP observed in the *osbadh1 osbadh2* double mutant suggests that *OsBADH1*, although not the primary fragrance gene, contributes to aldehyde-associated buffering capacity when *OsBADH2* is disrupted. This interpretation is consistent with broader work on plant aldehyde dehydrogenases showing that these enzymes participate not only in specialized metabolism but also in detoxification and stress-related homeostasis [41,42]. Additional aldehyde dehydrogenase family members may also contribute to metabolic compensation and stress adaptation, warranting further investigation.

A second major implication of the present results is that enhancement of aroma was tightly associated with impairment of GABA-linked stress protection. The double mutant displayed the lowest endogenous GABA content together with the most severe physiological stress injury, including reduced survival, lower relative water content and chlorophyll content, and increased electrolyte leakage, MDA content, and ROS accumulation (Figure 3 and Figure 4; Supplementary Tables S4 and S5). These physiological changes indicate that the double mutant experienced greater membrane damage and oxidative stress-associated injury under abiotic stress conditions. Although ion homeostasis was not directly examined in the present study, future analyses of Na^+^, K^+^, and Na^+^/K^+^ ratios may provide additional mechanistic insight into the enhanced salt sensitivity observed in the *osbadh1 osbadh2* double mutant. Similarly, antioxidant enzyme activities were not directly measured; however, the increased ROS accumulation, MDA content, and electrolyte leakage observed in the double mutant suggest impaired oxidative stress homeostasis. Future analyses of antioxidant enzyme activities, including SOD, CAT, APX, and POD, will help further clarify the mechanisms underlying oxidative stress imbalance in the double mutant. This stress-sensitive phenotype is biologically meaningful because GABA is widely recognized as a multifunctional metabolite involved in osmotic adjustment, intracellular pH regulation, antioxidant defense, carbon–nitrogen balance, and stress signaling [34,35,36,43]. Earlier work in fragrant rice also showed that salt treatment can reshape the relationships among 2-AP, proline, and GABA and that fragrance-associated backgrounds may show reduced performance under salt- or drought-related stress [37,39,40,43]. Taken together, our data indicate that the enhanced aroma phenotype in the double mutant is coupled to weakened GABA-associated buffering capacity under abiotic stress.

This interpretation is further supported by metabolite profiling data showing reduced levels of glutamate, proline, and γ-aminobutyraldehyde together with increased 2-AP accumulation in the *osbadh1 osbadh2* double mutant (Supplementary Table S4). These metabolite patterns support a shift in metabolic balance from GABA-associated pathways toward enhanced aroma-associated 2-AP biosynthesis. Future rescue experiments using exogenous GABA application will be valuable for directly testing whether reduced GABA accumulation contributes causally to the stress-sensitive phenotype of the *osbadh1 osbadh2* double mutant. The molecular analyses further support this interpretation. KEGG pathway-based analysis of the targeted stress-associated gene expression and metabolite profiling datasets indicated that the affected pathways were mainly related to glutamate metabolism, alanine, aspartate and glutamate metabolism, arginine and proline metabolism, the GABA shunt, plant hormone signal transduction, and MAPK signaling (Figure 5A). In parallel, the heatmap and targeted expression analysis showed that the strongest transcriptional shift occurred in the double mutant, including downregulation of *OsGAD1*, *OsGAD2*, *OsP5CS1*, *OsDREB2A*, *OsNAC6*, *OsCATB*, and *OsAPX1*, together with reduced expression of the edited target genes *OsBADH1* and *OsBADH2* in the corresponding mutant backgrounds (Figure 5B and Figure 6). These coordinated changes suggest that simultaneous loss of the two BADH paralogs affects not only volatile metabolism but also broader transcriptional networks associated with GABA homeostasis, ROS control, and stress adaptation. This interpretation agrees with recent models positioning GABA metabolism as an integrated stress-response system rather than an isolated branch pathway [34,35,36,41,42].

**Figure 7:**
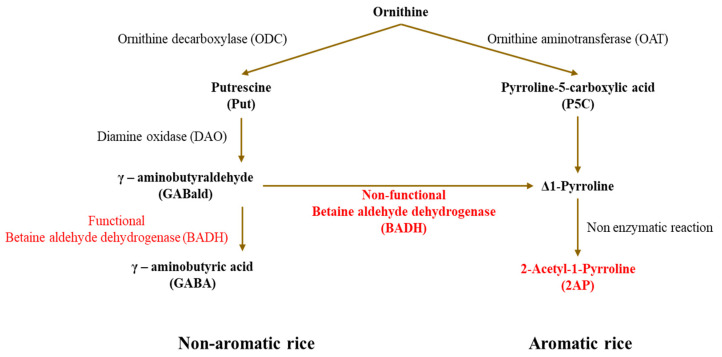
Simplified model of BADH-dependent metabolic flux toward GABA or 2-AP in rice. Functional BADH favors GABA production from γ-aminobutyraldehyde, whereas non-functional BADH promotes flux toward 2-AP accumulation.

The conceptual model presented in Figure 7 provides a framework for integrating these observations. Functional BADH activity favors metabolic conversion of γ-aminobutyraldehyde toward GABA formation, whereas impaired BADH function promotes the accumulation of Δ^1^-pyrroline-related intermediates and downstream 2-AP production [2,8,9]. Within this framework, the phenotype of the *osbadh1 osbadh2* double mutant can be interpreted as a more pronounced loss of BADH-dependent aldehyde-buffering capacity, resulting in reduced GABA accumulation and enhanced 2-AP formation. Therefore, BADH-centered genome editing remains an effective route for fragrance improvement, but our results indicate that stronger disruption of BADH-associated metabolism may also weaken performance in salinity- and drought-prone environments [37,40,43]. From a breeding perspective, this finding extends previous studies on fragrant rice mutants and cultivars carrying natural *badh2* alleles, which have mainly emphasized aroma enhancement through increased 2-AP accumulation. Our results suggest that combined disruption of *OsBADH1* and *OsBADH2* may further improve fragrance but also introduce a physiological trade-off related to stress-associated metabolic homeostasis. Future multi-environment field evaluations will be necessary to determine the stability of aroma-associated traits and stress responses under diverse cultivation conditions. In addition, although GC–MS-based 2-AP quantification is widely used as a biochemical indicator of rice fragrance, sensory evaluation will be important for linking 2-AP accumulation with consumer-perceived aroma intensity. Overall, this study expands the current understanding of rice BADH function by showing that *OsBADH1* and *OsBADH2* are linked not only to aroma-associated 2-AP accumulation but also to GABA-related metabolic homeostasis and abiotic stress resilience.

## 5. Conclusions

Simultaneous disruption of *OsBADH1* and *OsBADH2* enhanced aroma-associated metabolism but weakened GABA-linked abiotic stress tolerance in rice. The *osbadh1 osbadh2* double mutant accumulated more 2-acetyl-1-pyrroline (2-AP) than the *osbadh2* single mutant, indicating that *OsBADH1*, although not the primary fragrance gene, contributes to BADH-associated metabolic homeostasis. In parallel, the double mutant showed the lowest GABA content and the strongest sensitivity to salinity and drought stress, while no major defects in growth or agronomic traits were detected under normal conditions. KEGG enrichment, heat map analysis, and targeted expression profiling further showed that combined loss of *OsBADH1* and *OsBADH2* disturbed GABA-associated metabolic and stress-responsive pathways. Together, these findings reveal a clear trade-off between enhanced aroma and reduced stress resilience in rice with combined BADH disruption. This study expands the current understanding of rice BADH function by showing that *OsBADH1* and *OsBADH2* are linked not only to 2-AP accumulation but also to metabolic buffering and abiotic stress adaptation, providing a useful framework for balancing grain quality and stress tolerance in fragrance-oriented rice breeding.

## Figures and Tables

**Table 1:** Vegetative and agronomic traits of the wild type, *osbadh1*, *osbadh2*, and *osbadh1 osbadh2* double mutant under non-stress conditions.

Genotype	Plant Height (cm)	Tiller Number	Panicle Length (cm)	Primary Branches	Secondary Branches	Grain Number Per Panicle	Seed Setting Rate (%)	1000-Grain Weight (g)
WT	102.5 ± 4.3	12.1 ± 1.2	23.4 ± 1.1	11.2 ± 0.9	22.6 ± 1.8	128.6 ± 8.7	92.4 ± 2.1	24.8 ± 0.9
*osbadh1*	101.8 ± 5.0	11.8 ± 1.4	23.1 ± 1.3	10.9 ± 1.0	21.8 ± 2.0	125.9 ± 7.9	91.6 ± 2.3	24.5 ± 1.0
*osbadh2*	100.6 ± 4.7	11.5 ± 1.3	22.9 ± 1.2	10.7 ± 0.8	21.2 ± 1.7	122.3 ± 9.1	90.8 ± 2.5	24.2 ± 0.8
*osbadh1* × *osbadh2*	99.8 ± 5.2	11.3 ± 1.5	22.7 ± 1.4	10.5 ± 0.9	20.9 ± 1.9	120.7 ± 8.5	89.9 ± 2.7	24.0 ± 0.9

Data are presented as mean ± standard deviation (SD) from at least three biological replicates. No statistically significant differences were observed among genotypes (*p* > 0.05, one-way ANOVA).

## Data Availability

The original contributions presented in the study are included in the article/Supplementary Materials; further inquiries can be directed to the corresponding author.

## Supplementary Materials

The following supporting information can be downloaded at https://www.mdpi.com/article/10.3390/genes17050579/s1, Figure S1. CRISPR/Cas9 construct design, rice transformation, and mutation screening of *OsBADH1* and *OsBADH2*; Figure S2. Predicted frameshift mutations and premature termination caused by representative *osbadh1* and *osbadh2* edited alleles; Figure S3. Representative agronomic phenotypes and T-DNA segregation analysis of WT, *osbadh1*, *osbadh2*, and *osbadh1 osbadh2* plants under normal growth conditions; Table S1. sgRNA design information and mismatch-based off-target prediction results for CRISPR/Cas9-mediated editing of *OsBADH1* and *OsBADH2*; Table S2. The primers list used in this study; Table S3. Raw data for 2-acetyl-1-pyrroline (2-AP) quantification; Table S4. Metabolite profiling associated with GABA and 2-acetyl-1-pyrroline (2-AP) biosynthesis pathways in WT, *osbadh1*, *osbadh2*, and *osbadh1 osbadh2* double mutants; Table S5. Additional physiological measurements under salinity and drought stress; Table S6. Mutation types identified in CRISPR/Cas9-edited OsBADH1 and OsBADH2 lines; Table S7. Segregation analysis of *osbadh1*, *osbadh2*, and *osbadh1 osbadh2* double mutants in the F2 population derived from a cross between single mutants.
