# Supplementary material for: OsBADH1–OsBADH2 Double Mutants Increase 2-Acetyl-1-Pyrroline Accumulation and Alter GABA-Associated Abiotic Stress Responses in Rice
Source: Genes (Basel). 2026 May 18;17(5):579. doi: 10.3390/genes17050579 (PMC13205729; doi:10.3390/genes17050579)
Supplement: Supplementary file 1 [file genes-17-00579-s001.zip › genes-4305140-supplementary.pdf]

Supplementary Data

OsBADH1–OsBADH2 Double Mutants Increase 2-Acetyl-1-Pyrroline Accumulation and Alter GABA-Associated Abiotic Stress Responses in Rice

Yu-Jin Jung[1,2], Jin-Young Kim[1] and Kwon Kyoo Kang [1,2*]

Contents

Supplementary Figure S1. CRISPR/Cas9 construct design, rice transformation, and mutation screening of *OsBADH1* and *OsBADH2*.

Supplementary Figure S2. Predicted frameshift mutations and premature termination caused by representative *osbadh1* and *osbadh2* edited alleles.

Supplementary Figure S3. Representative agronomic phenotypes and T-DNA segregation analysis of WT, *osbadh1*, *osbadh2*, and *osbadh1 osbadh2* plants under normal growth conditions.

Supplementary Table S1. sgRNA design information and mismatch-based off-target prediction results for CRISPR/Cas9-mediated editing of *OsBADH1* and *OsBADH2*.

Supplementary Table S2. The primers list used in this study.

Supplementary Table S3. Raw data for 2-AP quantification.

Supplementary Table S4. Metabolite profiling associated with GABA and 2-AP biosynthesis pathways in WT, *osbadh1*, *osbadh2*, and *osbadh1 osbadh2* double mutants.

Supplementary Table S5. Additional physiological measurements under salinity and drought stress.

Supplementary Table S6. Mutation types identified in CRISPR/Cas9-edited *OsBADH1* and *OsBADH2* lines.

Supplementary Table S7. Segregation analysis of *osbadh1*, *osbadh2*, and *osbadh1 osbadh2* double mutants in the F_2 population derived from a cross between single mutants.

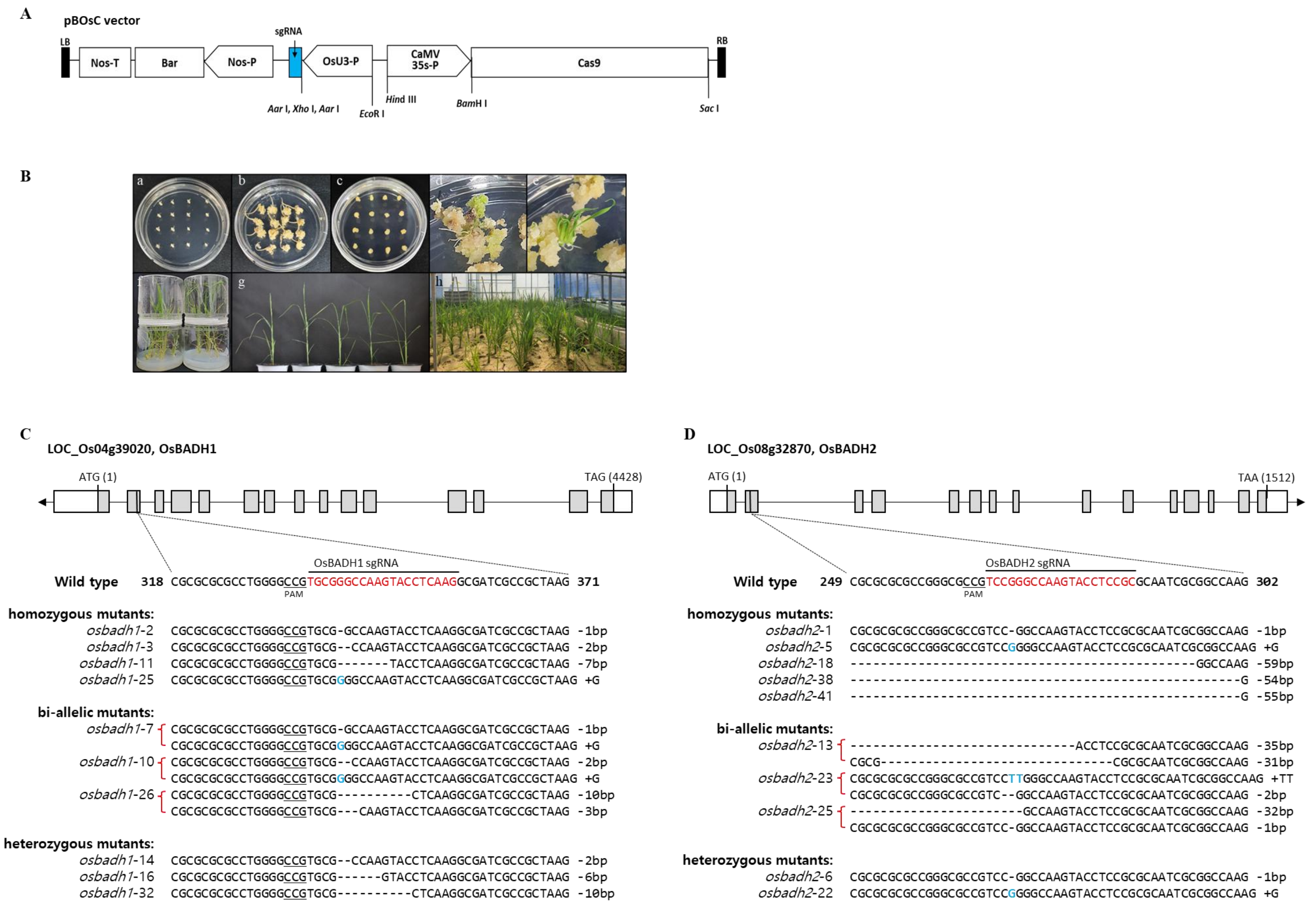

Supplementary Figure S1. CRISPR/Cas9 construct design, rice transformation, and mutation screening of *OsBADH1* and *OsBADH2*. (A) Schematic representation of the pBOsC CRISPR/Cas9 binary vector carrying sgRNAs targeting *OsBADH1* or *OsBADH2* under the control of the rice U3 promoter. (B) Induction of embryogenic calli from mature seeds of rice cv. Dongjin and regeneration procedure used for Agrobacterium-mediated transformation. (C) Representative PCR and deep sequencing results used for screening edited plants at the *OsBADH1* and *OsBADH2* target loci.

A **OsBADH1**

```
 -1bp   1   MAAPSAIPRRGLFIGGGWREPSLGRRLPVVNPATEATIGDIPAATAEDVELAVSAARDAF         60
 -2bp   1   MAAPSAIPRRGLFIGGGWREPSLGRRLPVVNPATEATIGDIPAATAEDVELAVSAARDAF         60
 -3bp   1   MAAPSAIPRRGLFIGGGWREPSLGRRLPVVNPATEATIGDIPAATAEDVELAVSAARDAF         60
 -6bp   1   MAAPSAIPRRGLFIGGGWREPSLGRRLPVVNPATEATIGDIPAATAEDVELAVSAARDAF         60
 -7bp   1   MAAPSAIPRRGLFIGGGWREPSLGRRLPVVNPATEATIGDIPAATAEDVELAVSAARDAF         60
-10bp   1   MAAPSAIPRRGLFIGGGWREPSLGRRLPVVNPATEATIGDIPAATAEDVELAVSAARDAF         60
   +G   1   MAAPSAIPRRGLFIGGGWREPSLGRRLPVVNPATEATIGDIPAATAEDVELAVSAARDAF         60
   WT   1   MAAPSAIPRRGLFIGGGWREPSLGRRLPVVNPATEATIGDIPAATAEDVELAVSAARDAF         60
            ************************************************************

 -1bp  61   GRDGGRHWSRAPGAVRPSTSRRSPLRLKIRNL----------------------------         92
 -2bp  61   GRDGGRHWSRAPGAVRQVPQGDRR------------------------------------         84
 -3bp  61   GRDGGRHWSRAPGAVRKYLKAIAAKIKDKKSYLALLETLDSGKPLDEAAGDMEDVAACFE ……….. 504
 -6bp  61   GRDGGRHWSRAPGAVRYLKAIAAKIKDKKSYLALLETLDSGKPLDEAAGDMEDVAACFEY ……….. 503
 -7bp  61   GRDGGRHWSRAPGAVRTSRRSPLRLKIRNLI-----------------------------         91
-10bp  61   GRDGGRHWSRAPGAVRSRRSPLRLKIRNLI------------------------------         90
   +G  61   GRDGGRHWSRAPGAVRGQVPQGDRR-----------------------------------         85
   WT  61   GRDGGRHWSRAPGAVRAKYLKAIAAKIKDKKSYLALLETLDSGKPLDEAAGDMEDVAACF ……….. 505
            ****************
```

B **OsBADH2**

```
   +G   1   MATAIPQRQLFVAGEWRAPALGRRLPVVNPATESPIGEIPAGTAEDVDAAVAAAREALKR         60
-32bp   1   MATAIPQRQLFVAGEWRAPALGRRLPVVNPATESPIGEIPAGTAEDVDAAVAAAREALKR         60
-35bp   1   MATAIPQRQLFVAGEWRAPALGRRLPVVNPATESPIGEIPAGTAEDVDAAVAAAREALKR         60
 -2bp   1   MATAIPQRQLFVAGEWRAPALGRRLPVVNPATESPIGEIPAGTAEDVDAAVAAAREALKR         60
-55bp   1   MATAIPQRQLFVAGEWRAPALGRRLPVVNPATESPIGEIPAGTAEDVDAAVAAAREALKR         60
  +TT   1   MATAIPQRQLFVAGEWRAPALGRRLPVVNPATESPIGEIPAGTAEDVDAAVAAAREALKR         60
 -1bp   1   MATAIPQRQLFVAGEWRAPALGRRLPVVNPATESPIGEIPAGTAEDVDAAVAAAREALKR         60
-31bp   1   MATAIPQRQLFVAGEWRAPALGRRLPVVNPATESPIGEIPAGTAEDVDAAVAAAREALKR         60
   WT   1   MATAIPQRQLFVAGEWRAPALGRRLPVVNPATESPIGEIPAGTAEDVDAAVAAAREALKR         60
            ************************************************************

   +G  61   NRGRDWARAPGAVRGQVPPRNRGQDNREEI------------------------------         90
-32bp  61   NRGRQ-----------VPPRNRGQDNREEI------------------------------         79
-35bp  61   NRGR------------EPPRNRGQDNREEI------------------------------         78
 -2bp  61   NRGRDWARAPGAVG-QVPPRNRGQDNREEI------------------------------         89
-55bp  61   NRGRD-------------------------------------------------------         65
  +TT  61   NRGRDWARAPGAVLGPSTSAQSRPR-----------------------------------         85
 -1bp  61   NRGRDWARAPGAVR-PSTSAQSRPR-----------------------------------         84
-31bp  61   NRGRDWARAQ-----------SRPR-----------------------------------         74
   WT  61   NRGRDWARAPGAVRAKYLRAIAAKIIERKSELARLETLDCGKPLDEAAWDMDDVAGCFEY ……….. 120
            ****
```

Supplementary Figure S2. Predicted frameshift mutations and premature termination caused by representative *osbadh1* and *osbadh2* edited alleles. Alignment of wild-type and edited nucleotide sequences showing the representative 10-bp deletion in *OsBADH1* and 55-bp deletion in *OsBADH2* selected for further analysis. Predicted frameshift effects and premature stop codons are indicated.

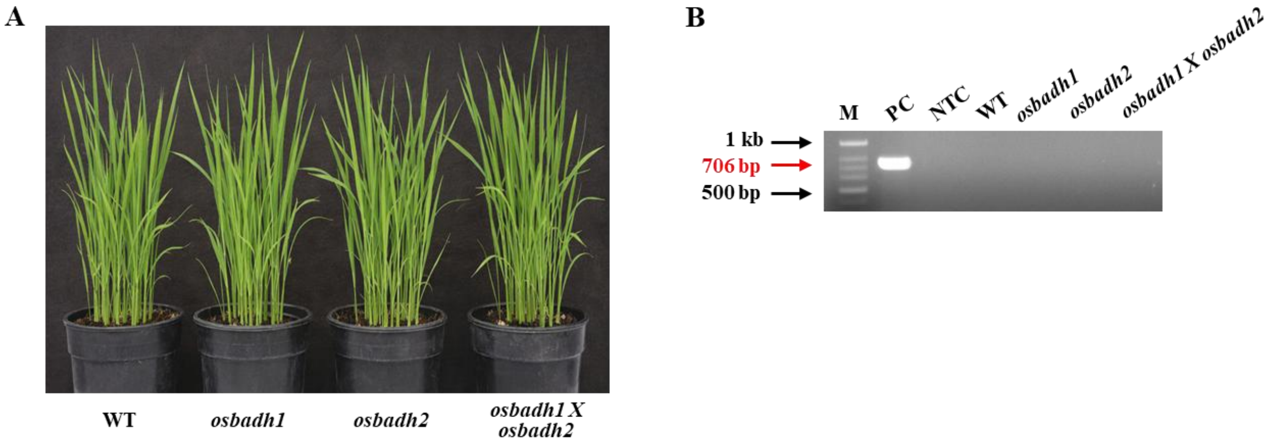

Supplementary Figure S3. Representative agronomic phenotypes and T-DNA segregation analysis of WT, *osbadh1*, *osbadh2*, and *osbadh1 osbadh2* plants under normal growth conditions. PCR amplification using T-DNA-specific primers was performed to confirm segregation of the CRISPR/Cas9 construct in selected later-generation mutant lines. The expected T-DNA-specific amplicon size was 706 bp. WT, wild type.

Supplementary Table S1. sgRNA design information and mismatch-based off-target prediction results for CRISPR/Cas9-mediated editing of *OsBADH1* and *OsBADH2*.

Gene	sgRNA Target (5' to 3')	Cleavage Position (%)	Direction	GC Contents	Out-of-frame Score	Mismatches			
						0	1	2	3
OsBADH1	CTTGAGGTACTTGGCCCGCACGG	78.2	-	60	68.3	1	0	0	0
OsBADH2	GCGGAGGTACTTGGCCCGGACGG	77.2	-	70	62.1	1	0	0	0

Supplementary Table S2. The primers list used in this study.

Primer name	Sequence (primer direction 5′-3′)	purpose
J67 pBOsC sgSEQ - FW	CAGCTTGGCTCTAGTCGACC	Vector construction
K20 RGEN scaaffold RV	CGGTGCCACTTTTTCAAGTT	
*OsBADH1*_sgRNA up	ggcagCTTGAGGTACTTGGCCCGCA	
*OsBADH1*_sgRNA down	aaacTGCGGGCCAAGTACCTCAAGc	
*OsBADH2*_sgRNA up	ggcagGCGGAGGTACTTGGCCCGGA	
*OsBADH2*_sgRNA down	aaacTCCGGGCCAAGTACCTCCGCc	
T-DNA confirm-Nos ter Fw	TTGCGCGCTATATTTTGTTTT	T-DNA confirm
T-DNA confirm-Bar R Rv	CGTCAACCACTACATCGAGA	
*OsBADH1*_sgRNA 1st F1	CCCAACCGGAAGCCATGG	deep-sequencing
*OsBADH1*_sgRNA 1st R1	GTTTACGGCACTAAGAGCGT	
*OsBADH1*_sgRNA 2nd F1	acactctttccctacacgacgctcttccgatctgATCGGTGAAGGTGACATCCC	
*OsBADH1*_sgRNA 2nd R1	gtgactggagttcagacgtgtgctcttccgatctcAGGTTAGCAGCATAGGAGAGC	
*OsBADH2*_sgRNA 1st F1	CATCGGTACCCTCCTCTTCA	
*OsBADH2*_sgRNA 1st R1	GGGGGCTTATACCGAAGCTA	
*OsBADH2*_sgRNA 2nd F1	acactctttccctacacgacgctcttccgatctgTTTGCGTGTCGGTGCGCA	
*OsBADH2*_sgRNA 2nd R1	gtgactggagttcagacgtgtgctcttccgatctcCTGTACGGAACACACGCAC	

Supplementary Table S3. Raw data for 2-AP quantification.

Genotype	Replicate 1	Replicate 2	Replicate 3	Mean (mg kg^{-1} DW)	SD
WT	4.8	5.5	5.3	5.2	0.36
osbadh1	5.2	5.7	5.6	5.5	0.26
osbadh2	27.1	29.5	28.6	28.4	1.20
Double	34.2	38.1	37.8	36.7	2.12

Raw GC–MS peak area values, standard-based quantification results, and calculated 2-AP contents for the wild type, *osbadh1*, *osbadh2*, and *osbadh1 osbadh2* double mutant.

Supplementary Table S4. Metabolite profiling associated with GABA and 2-AP biosynthesis pathways in WT, *osbadh1*, *osbadh2*, and *osbadh1 osbadh2* double mutants.

**Metabolite**	**WT (μmol g⁻¹ FW)**	***osbadh1***	***osbadh2***	***osbadh1 x osbadh2***	**Fold change (Double mutants vs WT)**
Glutamate	15.2 ± 1.3	14.8 ± 1.1	12.6 ± 1.0	11.9 ± 0.9	0.78
GABA	12.3 ± 1.2	11.8 ± 1.0	8.4 ± 0.9	5.6 ± 0.7	0.46
Proline	6.8 ± 0.6	6.5 ± 0.5	5.2 ± 0.4	4.7 ± 0.4	0.69
γ-aminobutyraldehyde	3.4 ± 0.4	3.1 ± 0.3	2.2 ± 0.3	1.6 ± 0.2	0.47
2-AP	5.2 ± 0.8	5.5 ± 0.9	28.4 ± 2.1	36.7 ± 2.8	7.06
Pyruvate	9.1 ± 0.7	8.9 ± 0.8	7.6 ± 0.6	7.1 ± 0.5	0.78
Alanine	4.3 ± 0.5	4.1 ± 0.4	3.5 ± 0.3	3.2 ± 0.3	0.74

Raw HPLC-based quantification data and calculated GABA contents for the wild type, *osbadh1*, *osbadh2*, and *osbadh1 osbadh2* double mutant.

Supplementary Table S5. Additional physiological measurements under salinity and drought stress.

Genotype	Relative water content (%)	Chlorophyll content (SPAD)	Electrolyte leakage (%)	MDA content (nmol g^{-1} FW)	ROS level (relative units)
WT	82.6 ± 3.5	38.4 ± 2.1	18.2 ± 2.3	2.8 ± 0.3	1.0
osbadh1	79.8 ± 3.9	37.2 ± 2.4	20.1 ± 2.6	3.1 ± 0.4	1.1
osbadh2	68.7 ± 3.1	32.5 ± 2.0	28.6 ± 3.0	4.5 ± 0.5	1.6
Double	55.3 ± 2.8	27.8 ± 1.9	41.2 ± 3.4	6.8 ± 0.6	2.3

Quantitative values of stress-related physiological parameters, including relative water content, chlorophyll content, electrolyte leakage, malondialdehyde (MDA), and ROS levels, in the wild type and mutant lines under abiotic stress conditions.

Supplementary Table S6. Mutation types identified in CRISPR/Cas9-edited *OsBADH1* and *OsBADH2* lines.

Target gene	No. of plants examined	No. of plants with mutations	Mutation rate (%)	Homozygous mutations		Bi-allele mutations		Heterozygous mutations	
				Number	%	Number	%	Number	%
OsBADH1	39	32	82.1	12	37.5	13	40.6	7	21.8
OsBADH2	42	35	83.3	17	48.6	14	40.0	4	11.4

Supplementary Table S7. Segregation analysis of *osbadh1*, *osbadh2*, and *osbadh1 osbadh2* double mutants in the F_2 population derived from a cross between single mutants.

Genotype	Expected ratio	Observed number	Expected number	χ^2 value	p-value
WT	1	24	25		
osbadh1	2	52	50		
osbadh2	2	49	50		
Double	1	25	25		
Total	—	150	150	0.16	0.98

Observed segregation ratios were compared with expected Mendelian ratios (1:2:2:1) using a chi-square (χ^2) test. No significant deviation from the expected ratio was detected ($p > 0.05$).
